# Direct interaction between GluR2 and GAPDH regulates AMPAR-mediated excitotoxicity

**DOI:** 10.1186/1756-6606-5-13

**Published:** 2012-04-26

**Authors:** Min Wang, Shupeng Li, Hongyu Zhang, Lin Pei, Shengwei Zou, Frank J S Lee, Yu Tian Wang, Fang Liu

**Affiliations:** 1 Department of Neuroscience, Centre for Addiction and Mental Health, Toronto, Canada; 2 Brain Research Center, University of British Columbia, Vancouver, Canada; 3 Department of Psychiatry, University of Toronto, Toronto, Canada; 4 Department of Neuroscience, Centre for Addiction and Mental Health, Clarke Division, 250 College Street, Toronto, ON, M5T 1R8, Canada

## Abstract

Over-activation of AMPARs (α−amino-3-hydroxy-5-methylisoxazole-4-propionic acid subtype glutamate receptors) is implicated in excitotoxic neuronal death associated with acute brain insults, such as ischemic stroke. However, the specific molecular mechanism by which AMPARs, especially the calcium-impermeable AMPARs, induce neuronal death remains poorly understood. Here we report the identification of a previously unrecognized molecular pathway involving a direct protein-protein interaction that underlies GluR2-containing AMPAR-mediated excitotoxicity. Agonist stimulation of AMPARs promotes GluR2/GAPDH (glyceraldehyde-3-phosphate dehydrogenase) complex formation and subsequent internalization. Disruption of GluR2/GAPDH interaction by administration of an interfering peptide prevents AMPAR-mediated excitotoxicity and protects against damage induced by oxygen-glucose deprivation (OGD), an in vitro model of brain ischemia.

## Introduction

Glutamate is the principal excitatory neurotransmitter in the brain and is involved in numerous physiological functions including neuronal circuit development, learning and memory [1]. Glutamate-induced neurotoxicity is implicated in neuropathological disorders such as stroke and epilepsy [2]. The effects of glutamate are mediated via two major subfamilies of ligand-gated ion channels: NMDAR (N-methyl-D-aspartate receptor) and AMPAR [3]. AMPAR mediates fast synaptic transmission at excitatory synapses, while NMDAR is critical in producing a number of different forms of synaptic plasticity [1]. In neurons, mature AMPA receptors are found as tetramers consisting of various combinations of GluR1 to GluR4 subunits [4], each of which has the same topology: three transmembrane domains and one membrain re-entrant loop. All subunits are permeable to both Na^+^ and Ca^2+^ ions with the exception of GluR2, which is uniquely impermeable to Ca^2+^. The majority of AMPA receptors *in vivo* contain GluR2 subunits whose ion selectivity is dominant over other subunits [5].

The accumulation of glutamate, which occurs immediately after ischemia, results in excessive stimulation of glutamate receptors and leads to neurotoxicity [6,7]. NMDAR-mediated neurotoxicity is dependent upon extracellular Ca^2+^ and is likely mediated by Ca^2+^ influx directly through receptor-gated ion channels [6,7]. AMPAR is also tightly associated with a selective pattern of neuronal loss in certain brain areas following both global and focal ischemia [8-20]. Similar to what is reported for NMDAR, excitotoxicity mediated by AMPAR lacking the GluR2 subunit is thought to be dependent on ion influx (Ca^2+^, Zn^2+^) through AMPAR channels following agonist stimulation [19-21]. However, as most native AMPARs in the hippocampus contain the GluR2 subunit and therefore are likely impermeable to Ca^2+^[22-26], it is still unclear how activation of the GluR2-containing AMPAR leads to neuronal cell death.

Protein-protein interactions with the AMPAR have been reported to affect function of AMPAR, among which the best characterized ones, such as GRIP (glutamate receptor interacting protein), ABP (AMPAR-binding protein), SAP97 (synapse-associated protein-97), PICK1 (protein interacting with C kinase-1), stargazin, NSF (N-ethylmaleimide-sensitive factor) and AP2 (adaptor protein-2) [27-34], bind to the intracellular carboxyl terminus of AMPAR. They regulate AMPAR function in a variety of ways, including modulation of AMPAR subcellular localization, clustering and/or trafficking. Recent studies have demonstrated that NARP (neuronal activity-regulated pentraxin) and N-cadherin interact with the amino terminus (NT) of AMPAR subunits and play an important role in AMPAR clustering [35] as well as dendritic spine formation [36]. In the present study, we have identified a new AMPAR-interacting partner, GAPDH. We show that secreted GAPDH binds specifically to the extracellular NT domain of the GluR2 subunit, a process which is promoted by AMPAR activation. Disruption of GluR2/GAPDH interaction prevents AMPAR-mediated excitotoxicity and protects against damage in OGD model.

## Results

### GluR2 subunit directly interacts with GAPDH via its Y142-K172 region of N-terminus

**Figure 1:**
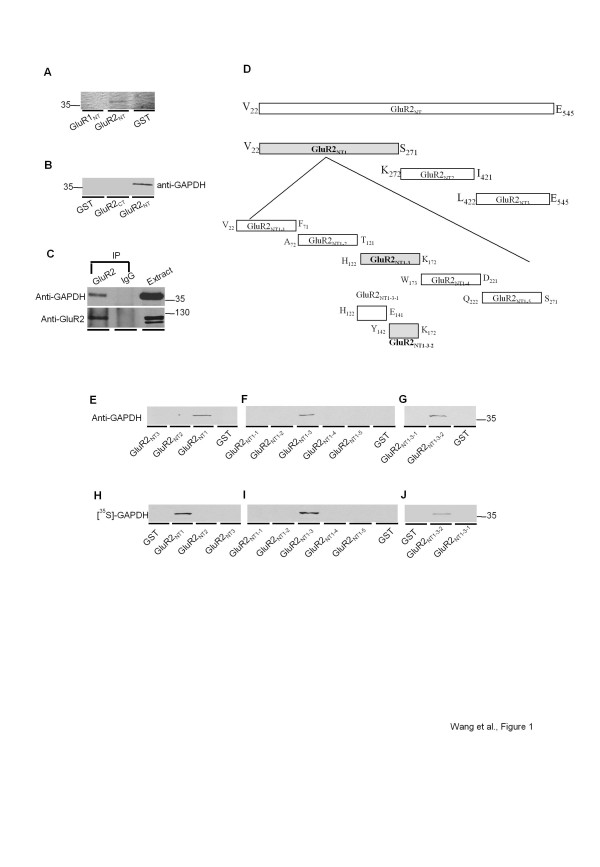
**Identification and characterization of GluR2/GAPDH interaction.*A,*** Coomassie blue stained SDS-PAGE gel of the protein(s) selectively affinity pulled down by GST-GluR2_NT_, GluR1_NT_ and GST alone from solubilized rat hippocampal lysates. Protein of interest: ~37 kDa. ***B,*** Western blot analysis of rat hippocampal proteins affinity purified by GST-GluR2_NT_, GST-GluR2_CT_ and GST from solubilized rat hippocampal lysates and immunoblotted with primary antibody against GAPDH. ***C,*** Co-immunoprecipitation of GAPDH by the GluR2 primary antibody from solubilized rat hippocampus. ***D,*** Schematic representation of GST-fusion proteins encoding truncated GluR2_NT_ segments. ***E-G,*** Western blot analysis of rat hippocampal proteins affinity purified by **(E)** GST-GluR2_NT1_, GST-GluR2_NT2_ GST-GluR2_NT3_ and GST; **(F)** GST-GluR2_NT1-1_, GST-GluR2_NT1-2_, GST-GluR2_NT1-3_, GST-GluR2_NT1-4_, GST-GluR2_NT1-5_ and GST; **(G)** GST-GluR2_NT1-3–1_, GST-GluR2_NT1-3–2_ and GST from solubilized rat hippocampal lysates and immunoblotted with primary antibody against GAPDH. ***H-J***: Using an i*n vitro* binding assay, [^35^ S]-GAPDH probe bound with GST-GluR2_NT1_**(H)**_,_ GST-GluR2_NT1-3_**(I)** and GST-GluR2_NT1-3–2_**(J)**, but not with other GST fusion proteins or GST alone.

To identify potential proteins that may interact with the NT domain of AMPAR subunits**,** we used GST-fusion proteins GST-GluR1_NT_ (A_19_-E_538_) and GST-GluR2_NT_ (V_22_-E_545_) to affinity “pull-down” proteins from solubilized rat hippocampal tissues along with GST alone as a control. The precipitated proteins were then identified by Coomassie brilliant blue staining following SDS-PAGE. A prominent protein band of ~37 kD was specifically precipitated by GST-GluR2_NT_, but not by GST alone or GST-GluR1_NT_ (Figure 1A). Mass spectrometry analysis (LC-MS/MS, Protana [now Transition Therapeutics]) of this protein band identified three fragments that were homologous to and covered 17% of the sequences within rat GAPDH (VIISAPSADAPMFVMGVNHEK; VIHDNFGIVEGLMTTVHAITATQK; VPTPNVSVVDLTCR). These results suggested that the GluR2 subunit might form a protein complex with GAPDH through its NT domain. We then confirmed the GluR2/GAPDH interaction with affinity purification experiments using GST-GluR2_NT_, GST-GluR2_CT_ (I_833_-I_883_) and GST alone. Subsequent Western blot analysis using a GAPDH antibody confirmed the association between GAPDH and GluR2_NT_, but not GluR2_CT_ (Figure 1B).

Before conducting further experiments, we examined whether GluR2/GAPDH complex exists *in vivo*. As shown in Figure 1C, the GluR2 antibody was able to co-immunoprecipitate (Co-IP) GAPDH from solubilized proteins extracted from rat hippocampal tissues confirming the *in vivo* association between GluR2 and GAPDH. In order to smooth the way for the following functional studies, three GluR2_NT_ GST-fusion proteins (GluR2_NT1_: V_22_-S_271_, GluR2_NT2_: K_272_-I_421_, GluR2_NT3_: L_422_-E_545_) were constructed (Figure 1D) and utilized in affinity purification experiments to delineate the region (s) of GluR2_NT_ involved in the interaction with GAPDH. As shown in Figure 1E, GST-GluR2_NT1_, but not GST-GluR2_NT2_, GST-GluR2_NT3_ or GST alone, precipitated GAPDH indicating that the GluR2 subunit interacts with GAPDH through its NT region V_22_-S_271_. A series of truncations of the GluR2_NT1_ region were then created to map the site that interacts with GAPDH (Figure 1D). As shown in Figure 1F and 1G, GST-GluR2_NT1-3_ (H_122_-K_172_) and GST-GluR2_NT1-3–2_ (Y_142_-K_172_) were able to precipitate GAPDH from rat hippocampal tissues.

While these results suggested the existence of the GluR2/GAPDH complex, it did not clarify whether this GluR2/GAPDH complex was formed through either a direct interaction or was mediated indirectly by other accessory binding proteins. Therefore we performed *in vitro* binding assays to examine whether GAPDH and the GluR2 subunit directly interact with each other. As shown in Figure 1H, *in vitro* translated [^35^ S]-GAPDH probe bound with GST-GluR2_NT1_ but not with GST-GluR2_NT2,_ GST-GluR2_NT3_ or GST alone, indicating the specificity of the direct protein-protein interaction between GAPDH and GluR2_NT1_. Consistent with the results from affinity purification experiments, the *in vitro* translated [^35^ S]-GAPDH probe only hybridized with GST-GluR2_NT1-3_ and GST-GluR2_NT1-3–2_, (Figure 1I, J). Together, these data provided *in vitro* evidence that GAPDH forms a direct protein-protein interaction with the GluR2 subunit through the Y_142_-K_172_ region of the GluR2_NT_.

### Agonist-facilitated GluR2/GAPDH complex formation occurs extracellularly

**Figure 2:**
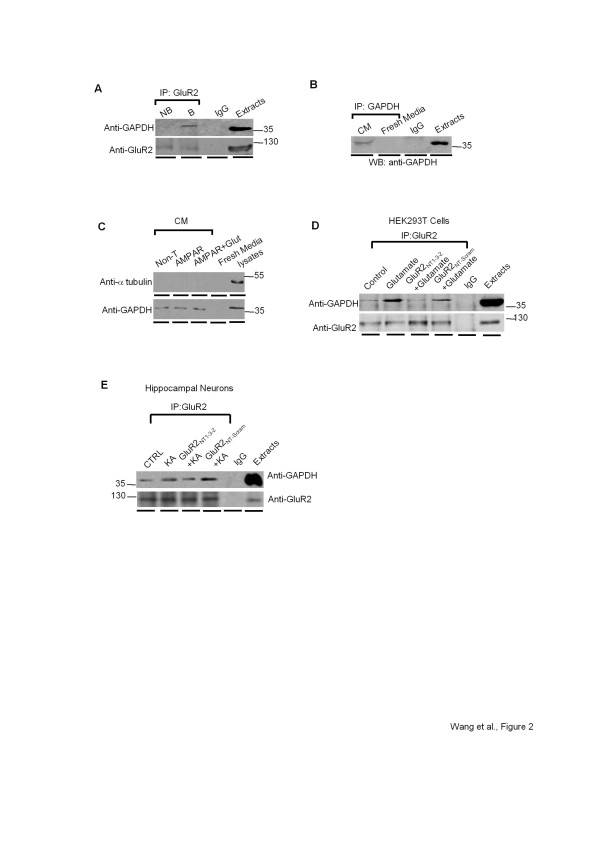
**GluR2/GAPDH interaction occurs extracellularly.*A,*** Rat hippocampal neurons were incubated with sulfo-NHS-LC biotin to label cell surface proteins. GAPDH that co-immunoprecipitated with GluR2 antibody was examined in both non-biotinylated (NB) and biotinylated (B) proteins. ***B,*** Using a rabbit anti-GAPDH antibody, GAPDH was immunoprecipitated from the conditioned medium (CM; medium incubated with neurons/cells for 24 hours) of primary cultures of rat hippocampus but not from fresh medium. A mouse GAPDH antibody was used for Western blotting and rabbit IgG was used as negative control. ***C,*** Western blot analysis of GAPDH and α-tubulin in concentrated conditioned medium of non-transfected HEK-293 T cells (non-T) and HEK-293 T cells transfected with GluR1/2 subunits (AMPAR), in the presence or absence of glutamate (AMPAR + Glut). Cell lysates were used as controls. ***D-E:*** Coimmunoprecipitation of GAPDH by primary antibody against GluR2 subunit (with or without glutamate treatment) from HEK-293 T cells expressing GluR1/2 subunits **(D)** and hippocampal neurons **(E)** pre-treated with GluR2_NT1-3–2_ or GluR2_NT1-3–2-scram_ peptides (top panels). Each Coimmunoprecipitation was in parallel with Western blot analysis of the directly immunoprecipitated proteins (bottom panels). All western blot analysis and co-immunoprecipitation assays in this figure are representative of at least 3 independent experiments.

As the NT region of GluR2 locates extracellularly, we then investigated whether the GluR2/GAPDH interaction occurs extracellularly by performing cell surface biotinylation experiments in primary culture of rat hippocampus, in which cell surface proteins of neurons were labeled with sulfo-NHS-LC-biotin. As shown in Figure 2A, the GluR2 antibody precipitated GAPDH from the biotinylated (B, cell surface) fraction, but failed to pull down GAPDH from the non-biotinylated (NB, intracellular) fraction, suggesting that the GluR2/GAPDH complex formation occurs extracellularly. Consistent with our findings, a previous study demonstrated that GAPDH was constitutively secreted into the extracellular space in several mammalian cell lines including HEK-293 T cells and neuro-2a cells [37]. We therefore speculated that GAPDH might be secreted into the extracellular space and form a protein complex with GluR2_NT_. To test our hypothesis, we first confirmed GAPDH secretion in our cell lines by immunoprecipitating GAPDH from the conditioned medium (incubation with neurons/cells for 24 hours) of hippocampal primary cultures with a primary antibody against GAPDH. As shown in Figure 2B, GAPDH was immunoprecipitated from conditioned medium, but not from fresh medium. To further exclude the possibility that the observed GAPDH in the conditioned medium resulted from cell lysis, conditioned media from non-transfected HEK-293 T cells and from cells expressing GluR1/2 subunits were collected, concentrated and examined by Western blot analyses using anti-GAPDH and anti-α-tubulin antibodies. As shown in Figure 2C, regardless of GluR1/2 subunit expression, GAPDH was detected from both conditioned media and cell lysates, whereas α-tubulin (a cytoplasmic protein marker) was only detected from cell lysates, indicating that the GAPDH found in the conditioned medium is secreted from cells and is not a contaminant due to cell lysis.

Furthermore, we examined the effect of the AMPAR activation on the formation of GluR2/GAPDH complex. By conducting Co-IP experiments, we found that AMPAR activation with either 100 μM glutamate in HEK-293 T cells expressing GluR1/2 subunits or 100 μM kainic acid (KA) in hippocampal neurons facilitated the GluR2/GAPDH complex formation by 75 ± 18% and 58 ± 11% (mean ± SEM, n = 3), respectively (Figure 2D, E; top panels). In each Co-IP experiment, 500 μg of protein were incubated in the presence of primary antibodies anti-GluR2 or rabbit IgG, and 50 μg of extracted protein was used as positive control. The level of directly immunoprecipitated GluR2 subunit was not significantly altered by the agonist stimulation (Figure 2D, E; bottom panels). If the GluR2_NT1-3–2_ region is essential for GluR2 to interact with GAPDH, application of the peptide encoding GluR2_NT1-3–2_ would disrupt the GluR2/GAPDH interaction by competing with GluR2 for GAPDH. As expected, pre-incubation of the GluR2_NT1-3–2_ peptide (10 μM, 1 hour), but not the scrambled GluR2_NT1-3–2_ peptide (GluR2_NT1-3-2Scram_), significantly inhibited the agonist-induced increase of the GluR2/GAPDH complex formation in transfected HEK-293 T cells (Figure 2D, 65 ± 8% decrease; mean ± SE, n = 3) and in hippocampal neurons (Figure 2E, 46 ± 6% decrease; mean ± SE, n = 3). The fact that extracellular application of the interfering GluR2_NT1-3–2_ peptide was able to disrupt the GluR2/GAPDH interaction further supports the notion that the GluR2/GAPDH complex formation occurs extracellularly.

### **Disruption of GluR2/GAPDH interaction inhibits AMPAR-mediated excitotoxicity**

**Figure 3:**
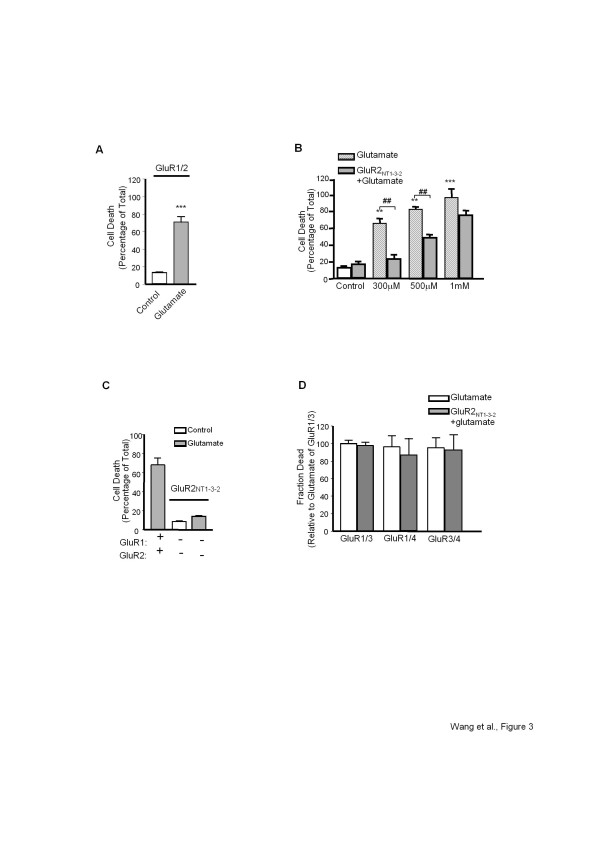
**Regulation of the AMPAR-mediated cell death in transfected cells.*A,*** Bar graph summarizing the quantitative measurements of PI fluorescence from HEK-293 T cells expressing GluR1/2 subunits with/without glutamate treatment (300 μM glutamate, 25 μM CTZ, 24 hr). ***Significantly different from control group (*P* < 0.001, n = 9 per group), t-test. ***B,*** Bar graph summarizing the quantitative measurements of PI fluorescence from HEK-293 T cells expressing GluR1/2 subunits with/without glutamate treatment at various doses in the presence/absence of GluR2_NT1-3–2_ peptide (10 μM, 1 hr). **, *** Significantly different from control group (*P* < 0.01, 0.001), ANOVA followed by *post-hoc* SNK test; ##, significant from the corresponding glutamate group (*P* < 0.01, n = 9 per group), t-test. ***C,*** Bar graph summarizing the quantitative measurements of PI fluorescence from non-transfected HEK-293 T cells or HEK-293 T cells expressing GluR1/2 subunits. Cells were pre-treated with the GluR2_NT1-3–2_ peptide with/without glutamate treatment (n = 9 per group). ***D,*** Bar graph summarizing the quantitative measurements of PI fluorescence from HEK-293 T cells expressing GluR1/3, GluR1/4 or GluR3/4 subunits with glutamate treatment in the presence/absence of the GluR2_NT1-3–2_ peptide (n = 9 per group). All PI fluorescence measurement assays were performed 3 times independently.

Both AMPAR and GAPDH have been independently shown to be involved in cell toxicity [38-42]. The observation that AMPAR activation promoted GluR2/GAPDH complex formation suggested that the GluR2/GAPDH interaction might be involved in AMPAR-mediated excitotoxicity. Before conducting further experiments, we first confirmed the ability of glutamate (300 μM, 24 hour; plus 25 μM cyclothiazide to prevent AMPAR desensitization) to induce cell death in HEK-293 T cells expressing GluR1/2 (Figure 3A), which is consistent with previous studies [43,44]. To investigate the role of the GluR2/GAPDH interaction in AMPAR-mediated cell death, HEK-293 T cells expressing GluR1/2 were pre-treated with the GluR2_NT1-3–2_ peptide (10 μM, 1 hour), which is able to disrupt the GluR2/GAPDH association (confirmed in Figure 2D). As shown in Figure 3B, pre-incubation with the GluR2_NT1-3–2_ peptide significantly attenuated glutamate-induced (300 μM, 500 μM) cell death. The GluR2_NT1-3–2_ peptide itself showed no effect in either the absence of glutamate treatment (Figure 3B) or in non-transfected cells regardless of glutamate treatment (Figure 3C). The specificity of the GluR2_NT1-3–2_ peptide was also confirmed in HEK-293 T cells expressing GluR1/3, GluR1/4 or GluR3/4 subunits, where pre-incubation with the GluR2_NT1-3–2_ peptide failed to inhibit AMPAR-mediated cell death (Figure 3D).

**Figure 4:**
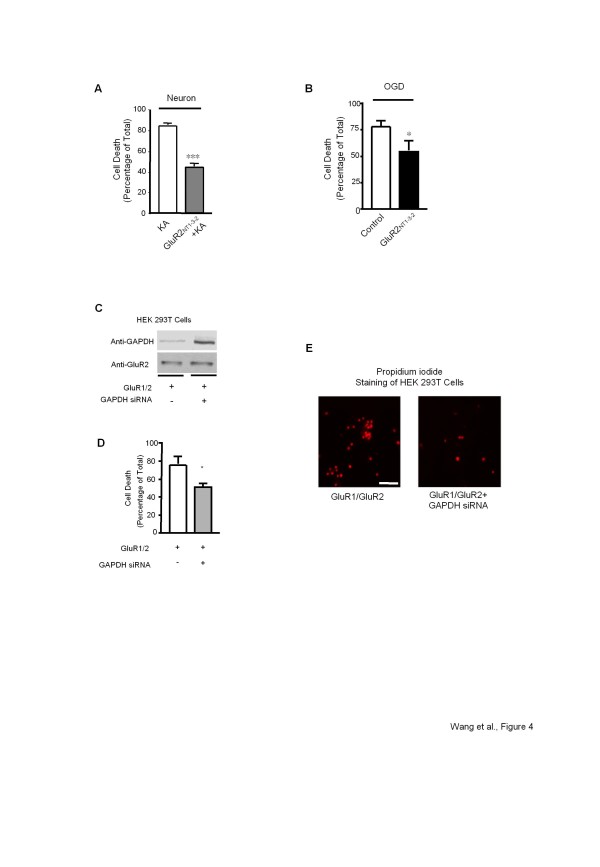
**Regulation of the AMPAR-mediated cell death in cultured neurons and OGD model.*A,*** Bar graph summarizing the quantitative measurements of PI fluorescence from rat hippocampal primary culture with KA treatment (100 μM, 1 hr) in the presence/absence of the GluR2_NT1-3–2_ peptide. ***Significantly different from KA group (*P* < 0.001, n = 9 per group), t-test. ***B,*** Bar graph summarizing the quantitative measurements of PI fluorescence from rat hippocampal primary culture with OGD protocol in the presence/absence of the GluR2_NT1-3–2_ peptide. *Significantly different from OGD group (*P* < 0.05, n = 9 per group), t-test. ***C,*** Western blot analysis of GAPDH (upper panel) and GluR2 (lower panel) expression in HEK 293 T cells expressing GluR1/2 subunits in the presence/absence of the GAPDH siRNA. ***D,*** Bar graph summarizing the quantitative measurements of PI fluorescence from HEK-293 T cells expressing GluR1/2 subunits with glutamate treatment in the presence/absence of the GAPDH siRNA (*P* < 0.05, n = 9 per group), t-test. ***E,*** Propidim iodide positive cells (red) from HEK-293 T expressing GluR1/2 subunits with glutamate treatment in the presence/absence of the GAPDH siRNA, scale bar 50 μm. All PI fluorescence measurement assays were performed 3 times independently.

To study the GluR2/GAPDH interaction in a relevant cellular milieu, rat hippocampal neurons were utilized in parallel experiments. We previously confirmed in Figure 2E that pre-incubating hippocampal neurons with the GluR2_NT1-3–2_ peptide interrupted the GluR2/GAPDH interaction promoted by the AMPAR activation. Thus, we examined whether the disruption of this interaction in hippocampal neurons by applying the GluR2_NT1-3–2_ peptide would rescue neurons from AMPAR-mediated excitotoxicity. AMPAR-mediated cell death was induced by treating neurons with KA (100 μM, 1 hour) in the presence of NMDAR and Ca^2+^ channel antagonists (10 μM MK-801 and 2 μM nimodipine). As shown in Figure 4A, pretreatment with the GluR2_NT1-3–2_ peptide significantly inhibited AMPAR-mediated cell death.

AMPAR-mediated toxicity is often considered a contributing, if not an underlying, causative factor in ischemia, which deprives brain cells of glucose and oxygen, causing irreversible brain damage within minutes. Cells in ischemic brain tissue undergo a number of changes: they rapidly lose their energy supplies, their membranes become depolarized, calcium loads are increased, reactive oxygen types are produced and excitotoxic effects are found. These biochemical changes are followed by irreversible changes to cellular structures and cell death. The oxygen glucose deprivation (OGD) cell lesion model represents a valid simulation of the conditions in brain ischemia [45,46]. Therefore, we assessed the effectiveness of the GluR2_NT1-3–2_ peptide to rescue cells from neurotoxic stress in the OGD model to verify the implication of the GluR2/GAPDH interaction in ischemia. As shown in Figure 4B, the GluR2_NT1-3–2_ peptide pretreatment (10 μM, 1 hour) was able to significantly attenuate OGD-induced cell death (30.4% ± 9.5%) in the presence of 10 μM MK-801 and 2 μM nimodipine.

In order to further confirm the role of GAPDH in the AMPAR-mediated cell death, GAPDH siRNA was transfected into HEK-293 T cells to block the expression of GAPDH, but not the expression of GluR2 (Figure 4C). As shown in Figure 4D-E, AMPAR-mediated cell death was significantly attenuated in the presence of GAPDH siRNA. Together, these data suggest that the GluR2/GAPDH interaction may play a critical role in the GluR2-contaning AMPAR-mediated cell death.

### Activation of AMPAR induces AMPAR/GAPDH complex internalization through the GluR2/GAPDH interaction

**Figure 5:**
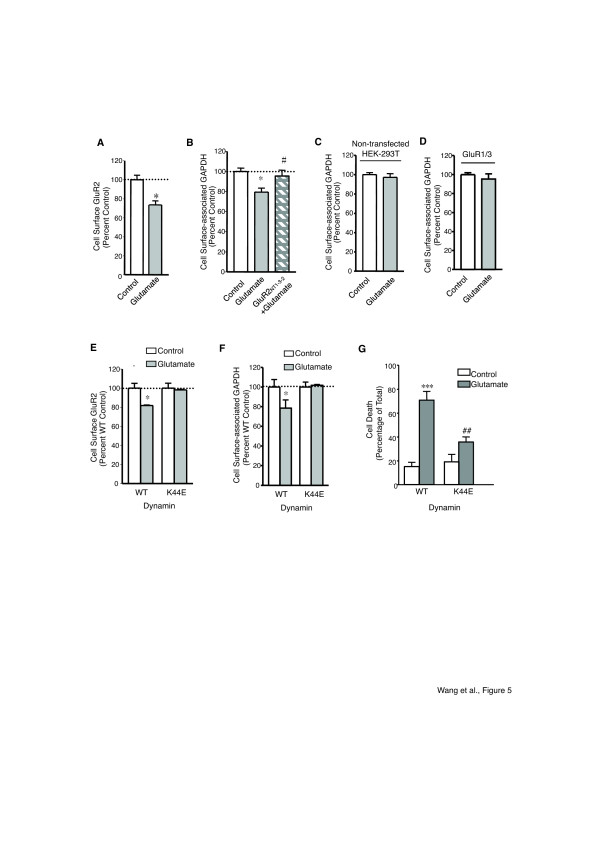
**Activation of AMPAR induces GluR2/GAPDH co-internalization.*A,*** Quantification of GluR2 expression at the plasma membrane with/without glutamate treatment (100 μM, 30 minutes) in HEK-293 T cells expressing GluR1/2 subunits. *Significantly different from control group (*P* < 0.05, n = 9 per group), t-test. ***B,*** Quantification of cell surface-associated GAPDH with/without glutamate treatment in the presence/absence of the GluR2_NT1-3–2_ peptide in HEK-293 T cells expressing GluR1/2 subunits. *Significantly different from control group; #, significantly different from glutamate group (*P* < 0.05, n = 9 per group), ANOVA followed by *post-hoc* SNK test. ***C,*** Quantification of cell surface-associated GAPDH in non-transfected HEK-293 T cells with/without glutamate treatment (n = 9 per group). ***D,*** Quantification of cell surface-associated GAPDH with/without glutamate treatment in HEK-293 T cells expressing GluR1/3 subunits (n = 9 per group). Quantification of plasma membrane GluR2 **(E)** or cell surface-associated GAPDH **(F)** expression at the plasma membrane with/without glutamate treatment in HEK-293 T cells expressing GluR1/2 subunits with wild type dynamin (WT) or mutant K44E dynamin (K44E). *Significantly different from the corresponding control group (*P* < 0.05, n = 9 per group), t-test. ***G,*** Bar graph summarizing the quantitative measurements of PI fluorescence from HEK-293 T cells expressing GluR1/2 subunits with wild type dynamin (WT) or mutant K44E dynamin (K44E) with/without glutamate treatment. ***Significantly different from control WT group (*P* < 0.001, n = 9 per group); ##significantly different from control K44E group (*P* < 0.01, n = 9 per group), t-test. All assays in this figure were performed 3 times independently.

Previous studies demonstrated that agonist stimulation could induce AMPAR endocytosis [47-49]. Thus, we examined whether the extracellular GAPDH would internalize along with AMPAR through the GluR2/GAPDH interaction upon the activation of AMPAR. To quantify GluR2 and GAPDH cell surface levels in HEK-293 T cells expressing GluR1/2, a cell-based ELISA assay was applied as previously described [49,50]. We first confirmed the results from previous studies that the glutamate stimulation (100 μM, 30 minutes) induced a significant decrease in plasma membrane GluR2 (Figure 5A). We then tested whether the cell surface-associated GAPDH is also decreased upon agonist stimulation of AMPAR. As shown in Figure 5B, activation of AMPAR significantly decreased the cell surface-associated GAPDH in HEK-293 T cells expressing GluR1/2, a phenomena that can be abolished by the pre-treatment of GluR2_NT1-3–2_ peptide. These data, together with the inability of glutamate stimulation to internalize the cell surface-associated GAPDH in the non-transfected HEK-293 T cells (Figure 5C) or HEK-293 T cells transfected with GluR1/3 subunits (Figure 5D), suggest that GAPDH internalization may be a passive process enabled by the GluR2/GAPDH interaction.

To further investigate whether the observed GAPDH internalization is dependent on the GluR2 internalization, we tested whether blockade of GluR2 endocytosis will inhibit GAPDH internalization. Previous studies demonstrated that GluR2 endocytosis is dynamin-dependent and that the expression of the dominant-negative dynamin mutant (K44E) was able to block the GluR2 internalization [47,49]. Thus, after confirming the ability of the K44E mutant to block the GluR2 internalization (Figure 5E), we examined whether the K44E mutant affected cell surface-associated GAPDH internalization in HEK-293 T cells expressing GluR1/2 subunits. As shown in Figure 5F, the K44E mutant significantly inhibited glutamate-induced cell surface-associated GAPDH internalization, indicating that GAPDH internalized through a dynamin-dependent pathway and further confirmed that GAPDH was co-internalize with the GluR2 subunit. Moreover, the K44E mutant also attenuated glutamate-induced cell death in HEK-293 T cells expressing GluR1/2 subunits (Figure 5G), indicating that GluR2/GAPDH complex internalization may play an important role in the GluR2-containing AMPAR-mediated cell death.

## Discussion

AMPAR-mediated excitotoxicity has been implicated in the pathogenesis of neuronal loss associated with a number of brain disorders, including transient forebrain ischemia [8-20]. However, the underlying mechanisms remain unclear. An uncontrollable rise in intracellular Ca^2+^ and Zn^2+^, with subsequent activation of diverse downstream cell death signals has been one of the most prominent hypotheses to explain excitotoxic neuronal death [19,20,51-55]. Although GluR2-containing AMPARs are calcium impermeable, recent studies have suggested that selective reductions in the expression of GluR2, resulting in an increase in Ca2 + −permeable AMPA receptors, have been associated with an increased vulnerability of neurons to ischemic injury [16,56-61]. Although the mechanisms involved are not fully understood, it has been suggested that GluR2 internalization may enhance the Ca^2+^-influx that results in neurotoxicity, either through newly synthesized Ca^2+^-permeable AMPARs [57] or by activation of a caspase-dependent apoptotic pathway [62]. Consistent with previous studies, our data has shown that agonist stimulation of AMPAR results in the internalization of GluR2 and promotes extracellular GAPDH internalization via a GluR2/GAPDH coupling-dependent process. This is the first evidence showing that the N-terminal of the GluR2 subunit plays an important role in AMPA receptor-mediated excitotoxicity through regulating AMPAR trafficking. Many studies have shown that agonist-induced GluR2 internalization is a dynamin-dependent process [47,49]. The observations of our study that mutant dynamin abolishes both GluR2 and GAPDH internalization and the inability of GAPDH to internalize in cells lacking GluR2 suggest that GAPDH internalization is a passive process facilitated by the GluR2/GAPDH interaction and mediated by GluR2 internalization.

Given the fact that GAPDH interacts with the extracellular NT of GluR2, it is likely that the GluR2/GAPDH protein complex may be in an endocytosed vesicle following the agonist-induced internalization. On this basis it would be logical to further ask how the GluR2/GAPDH complex gets out of the vesicle and promotes excitotoxic neuronal death. There are many possibilities for this question. First, the complex may be transported to the nucleus via a retrograde vesicle transport mechanism leading to the fusion of the vesicle with ER or nuclear membranes or via mechanisms recently proposed for the nuclear translocation of another plasma membrane receptor, the EGF receptor [63,64]. Second, the GluR2/GAPDH complex formation in the vesicle may lead to the activation of lysosome in the vesicle that breaks the vesicle and release the GluR2/GAPDH into the cytoplasm.

The possible mechanisms that underlie this GluR2/GAPDH related cell death is particularly interesting. It is somewhat surprising to find that the AMPAR-mediated cell death involves GAPDH, a key enzyme involved in glycolysis with a ubiquitous intracellular distribution. However, additional roles for GAPDH have been discovered recently, including membrane fusion/transport, binding to low molecular weight G proteins, regulation of the cytoskeleton, accumulation of glutamate into presynaptic vesicles, and apoptosis [65-71]. Recent studies have shown that GAPDH binds to Siah1 and triggers apoptosis [39]. Moreover GAPDH has also been reported to interact with p53 [72], a tumor suppressor and transcription factor that has been implicated in glutamate-mediated excitotoxicity [73-75]. Numerous evidence show that activation of p53 can trigger apoptosis (for reviews, see [76]) under conditions of cellular stress mediated by phosphorylation or acetylation of p53 [77]. Whether Siah1, p53 or other molecules are involved in GluR2/GAPDH-related cell death pathway requires much more additional work for a better understanding of the detailed molecular mechanisms.

Stroke is the second leading cause of death worldwide yet there are very few effective pharmacological treatments for patients suffering ischemic stroke. Thrombolytics such as alteplase and tenecteplase have been a significant advance in the treatment of ischemic stroke. However, thrombolytics must be given soon after a stroke to be effective (within 3 hours of ischemic episode). This short time frame has limited their use in many situations. There continues to be a significant unmet need for acute pharmacological treatments beyond thrombolytics. Advances in recent years include hypothermia [78-80], oxygen therapy [81], stem cell transplantation [82] and cerebral plasticity stimulation (trophic factor) strategies [83]. These novel techniques are intriguing, but will require further well-designed prospective trials to assess clinical feasibility, safety, and efficacy [84]. Another approach that has received considerable attention is agents that inhibit ischemia-induced excitotoxicity though directly blocking glutamate receptors. However, all have failed at various stages of development for a variety of reasons. One of the main drawbacks of the glutamate receptor antagonists is that they block normal excitatory neurotransmission necessary for maintaining basic brain functions. For this reason, much research has been directed at identifying drugs and peptides that may be able to selectively target protein-protein interactions that have more narrow function than a certain neurotransmitter receptor. In the present study, we have shown that administration of the interfering GluR2_NT1-3–2_ peptide to interrupt the GluR2/GAPDH interaction significantly mitigates neuronal cell death in a cell model of ischemia, revealing a previously unappreciated signaling pathway underlying AMPAR-mediated excitotoxicity and it may provide a new avenue for the development of a complementary therapeutics in the treatment of neuropathological disorders, such as stroke and epilepsy.

## Materials and methods

### Cell culture and transient transfection

HEK293 T cells were cultured in α-MEM (Invitrogen, Carlsbad, CA) supplemented with 10% fetal bovine serum (Invitrogen) and maintained in incubators at 37°C, 5% CO2. HEK293T cells were transiently transfected with plasmid constructs and/or siRNA using lipofectamine 2000 reagents (Invitrogen). Cells were harvested 48 hours post transfection.

### **Primary hippocampal neuron culture and OGD treatment**

Primary cultures from hippocampus were prepared from fetal Wistar rats (embryonic day 17–19) on Cell + (Sarstedt) culture dishes as previously described [85-87]. The cultures were used for experiments on 12–15 days after plating. Hippocampal cultures were pretreated GluR2_NT1-3–2_ peptides prior to kainic acid treatment. OGD treatment was performed in the presence of MK-801 and nimodipine as previously described [57].

### GST fusion proteins

To construct GST-fusion proteins encoding truncated GluR2 and GAPDH, cDNA fragments were amplified by using PCR method with specific primers. Except where specified, all 5′ and 3′ oligonucleotides incorporated BamH1 site (GGATCC) and Xho1 sites (CTCGAG), respectively, to facilitate subcloning into vector pGEX-4T3 (for GST-fusion protein construction). GST-fusion proteins were prepared from bacterial lysates with Glutathione Sepharose 4B beads as described by the manufacturer (Amersham). To confirm appropriate splice fusion and the absence of spurious PCR generated nucleotide errors, all constructs were resequenced.

### Protein affinity purification, *in vitro* binding, co-immunoprecipitation and western blot

Protein affinity purification, in vitro binding, co-immunoprecipitation and Western blot analyses were performed as previously described [85-87]. Antibodies used for immunoprecipitation, Western blots and cell surface ELISA assays include GAPDH (polyclonal from Abcam, monoclonal from Chemicon), GluR2 (Western blots: Chemicon; immunoprecipitation: Upstate), and α-tubulin (monoclonal, Sigma-Aldrich).

### Cell-ELISA assays

HEK-293 T cells transfected with plasmid constructs were treated with 100 μM glutamate or extracellular solution (ECS) before fixing in 4% (W/V) paraformaldehyde for 10 minutes in the absence (non-permeabilized conditions) or presence (permeabilized conditions) of 1% (V/V) Triton X-100. Cells were incubated in 1% (W/V) glycine for 10 minutes at 4°C to recover from the fixing. Cells were then incubated with specific primary antibodies for the purpose of labeling the receptors or proteins on the cell surface under non-permeabilized conditions or the entire receptor pool under permeabilized conditions. After incubation with corresponding HRP-conjugated secondary antibodies (Sigma-Aldrich), the HRP substrate o-phenylenediamine (Sigma-Aldrich) was added to produce a color reaction that was stopped with the equal volume of 3 N HCl. Fluorescence intensity in each well was measured with a plate reader (Victor3; PerkinElmer). The cell surface expression of HA-GluR2 after pre-treatment with glutamate was presented as the ratio of colorimetric readings under non-permeabilized conditions to those under permeabilized conditions, and then normalized to their respective control groups (pretreated with ECS). Afterwards, cells were scrapped from the dishes, and the protein concentration of each dish was measured. The results of cell surface expression of receptors or proteins were calibrated by the protein concentration of each well. Analysis was done using at least 9 separate wells in each group. Cell ELISA using primary hippocampal neurons was performed identically with assays using HEK-293 T cells, with the exception that the anti-GluR2 antibody (MAB397; Chemicon) was used as primary antibody instead of anti-HA.

### Quantification of AMPAR-mediated excitotoxicity

HEK-293 T cells transfected with GluR1/2 subunits were exposed to 300 μM glutamate/25 μM cyclothiazide at 37°C for 24 hour. Cells were allowed to recover for 24 hours at 37°C. To quantify AMPAR-mediated cell death, culture medium was replaced by extracellular solution containing 50 μg/ml of propidium iodide (PI) (Invitrogen, Carlsbad, CA). After 30 minutes incubation at 37°C, fluorescence intensity in each well was measured with a plate reader (Victor3; PerkinElmer, Waltham, MA). The fraction of dead cells was normalized to the total cell number. Primary hippocampal neurons were exposed to 100 μM KA/25 μM cyclothiazide in the presence of NMDAR and Ca^2+^ channel antagonists (10 μM MK-801 and 2 μM nimodipine, respectively) at 37°C for 1 hour.

### Cell biotinylation

For cell surface biotinylation, cells were rinsed four times with ice-cold PBS^2+^ (PBS containing 0.1 mM CaCl_2_ and 1.0 mM MgCl_2_) after treatment, and incubated twice with 1.0 mg/ml sulfo-NHS-LC-biotin (Pierce, Rockford, IL) for 20 minutes at 4°C. Non-reactive biotin was quenched by 20 minutes incubation at 4°C in ice-cold PBS^2+^ and 0.1 M glycine. Cells were solubilized in RIPA buffer (10 mM Tris, ph7.4, 150 mM NaCl, 1.0 mM EDTA, 0.1% (W/V) SDS, 1.0% (V/V) Triton X-100 and 1.0% (V/V) Sodium deoxycholate) containing protease inhibitors (1.0 mM PMSF and 1.0 μg/ml protease cocktail). Biotinylated and non-biotinylated proteins were separated from equal amounts of cellular protein by incubation with 50 μl of 50% slurry of immobilized streptavidin-conjugated beads (Pierce, Rockford, IL) overnight with constant mixing at 4°C. Unbound proteins (supernatant) were saved for later co-immunoprecipitation experiment. Proteins bound to streptavidin beads were eluted in biotin elution buffer. Biotinylated and non-biotinylated samples were applied to protein A/G PLUS-agarose (Santa Cruz Biotechnology, Santa Cruz, CA) for co-immunoprecipitation.

## Competing Interest

The authors declare that they have no competing interests.

## Authors’ contributions

MW carried out all experiments, with the help of SL for constructing GST-fusion proteins, HZ for AMAPR-mediated excitotoxicity assays, LP for the co-immunoprecipitation and SZ for the GST-pull down assays. FJK and YTW helped to edit the manuscript. FL supervised the study and wrote the manuscript. All authors read and approved the final manuscript.
